# Relationship between indexed aortic area and aortic diameter in bicuspid aortic valve aortopathy: A retrospective cohort study

**DOI:** 10.1016/j.amsu.2021.102342

**Published:** 2021-04-18

**Authors:** Metesh Acharya, Oswaldo Valencia, Mark Edsell, Maite Tome, Robert Morgan, Justin Nowell, Marjan Jahangiri

**Affiliations:** a Department of Cardiothoracic Surgery, St. George's Hospital, London, UK; b Department of Anaesthesia, St. George's Hospital, London, UK; c Department of Cardiology, St. George's Hospital, London, UK; d Department of Radiology, St. George's Hospital, London, UK

**Keywords:** Bicuspid aortic valve, Aneurysm, Aortic dissection, Aorta

## Abstract

**Background:**

Aortic dissection is a life-threatening complication of bicuspid aortic valve (BAV)-associated aortopathy. In these populations, whilst prophylactic replacement of proximal thoracic aortic aneurysms (TAAs) is generally recommended at threshold diameters ≥5.5 cm, dissection may occur in smaller aortas. An alternative size-based parameter, the cross-sectional aortic area/patient height ratio (indexed aortic area, IAA), correlates with increased dissection risk at abnormal values > 10 cm^2^/m. We sought to assess the utility of the IAA in identifying at-risk BAV-associated TAAs with abnormal IAA, albeit with sub-threshold aortic diameter.

**Materials and methods:**

We retrospectively identified 69 patients with BAV-associated TAAs who underwent surgical repair between 2010 and 2016. Aortic diameter was measured on pre-operative imaging, and IAA calculated, at the mid-sinus of Valsalva, sino-tubular junction and mid-ascending aorta for each patient. We determined proportions of aneurysms with IAA >10 cm^2^/m, median IAAs corresponding to aortic diameters <4.0 cm, 4.0–4.5 cm, 4.5–5.0 cm, 5.0–5.5 cm and >5.5 cm, and median aortic diameters corresponding to an abnormal IAA.

**Results:**

50.9%, 12.5% and 64.6% of aneurysms at the sinus of Valsalva, sino-tubular junction and mid-ascending aorta, respectively, had an abnormal IAA. 51.9% and 88.9% of patients with aortic diameter 4.5–5.0 cm and 5.0–5.5 cm, respectively, had an abnormal IAA. In aneurysms with abnormal IAA involving the sinus of Valsalva, sino-tubular junction, and mid-ascending aorta, median aortic diameters were 4.98 cm, 5.04 cm and 5.11 cm, respectively. Overall, 57/72 (79.2%) at-risk aneurysms with IAA >10 cm^2^/m had diameters smaller than the 5.5 cm guideline cut-off for surgical intervention.

**Conclusion:**

Significant proportions of BAV-associated TAAs are at increased risk of aortic dissection attending an IAA >10 cm^2^/m, whilst not fulfilling the size criteria indicating aortic surgery in contemporary guidelines. Further analysis of IAA in larger BAV cohorts is necessary to clarify its role in patient selection and optimal timing for prophylactic aortic replacement.

## Highlights

• Indexed aortic area is an alternative validated parameter of aortic dimensions.
• Significant proportions of aortic aneurysms associated with a bicuspid aortic valve have abnormal indexed aortic areas.
• Abnormal indexed aortic areas first emerge at diameters 4.5-5.0 cm, smaller than the 5.5 cm threshold for surgery.
• Many at-risk aneurysms may thus not meet size criteria for surgical intervention.
• In this series, over 79% of at-risk aneurysms had diameter <5.5 cm.

## Graphical abstract

**Figure:**
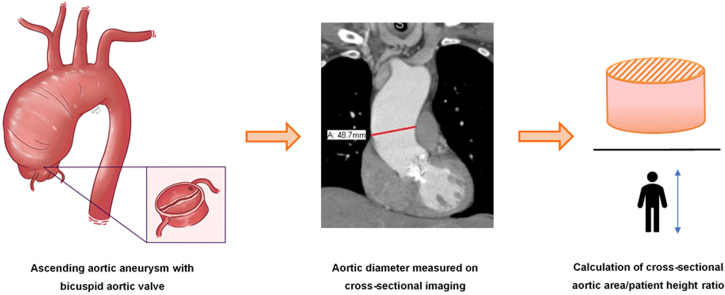

## 1 Introduction

Mounting evidence suggests acute dissection or rupture may occur within smaller aortas before achieving a 4.5–5.5 cm diameter, which would normally indicate surgical intervention in both tri-leaflet and bicuspid aortic valve (BAV) populations without inherited aortopathy or additional risk factors [[1], [2], [3]]. These criteria are unchanged in the updated 2020 American College of Cardiology/American Heart Association guidelines for the management of valvular heart disease [4]. However, in the International Registry of Acute Aortic Dissection series, 59% and 40% of patients presenting with acute type A dissection had ascending aortic diameter <5.5 cm and <5.0 cm, respectively [5]. Furthermore, 15% of patients with Marfan-related aortopathy undergo aortic dissection at diameters <5.0 cm [6]. Elastic tissue loss from the aortic media in BAV-associated aneurysms also renders them susceptible to dissection at risks comparable to those of a Marfan population [7,8]. It is also well-recognised that aortic aneurysms demonstrate accelerated expansion in the context of a BAV compared to a tri-leaflet valve, irrespective of valvular dysfunction [9].

A “one size fits all” approach to patient selection for prophylactic aortic surgery, heavily reliant on absolute diameter as a static determinant of aortic dimension, may thus be inappropriate, and unknowingly expose patients under routine surveillance to potentially life-threatening aortic complications. Pure diameter measurements fail to account for the irregular, elliptical shape of the thoracic aorta that is often encountered with BAVs [10].

An alternative size-based parameter, the cross-sectional aortic area/patient height ratio (indexed aortic area, IAA), has previously been validated in the stratification of mortality risk in patients with both bicuspid and tri-leaflet aortic valves and concomitant aortopathy. An abnormal ratio exceeding 10 cm^2^/m denotes an augmented dissection/rupture risk [6,8,[10], [11], [12]]. This cut-off value has been incorporated into the most recent iterations of the surgical guidelines as an indication for elective aortic replacement in patients with BAV aortopathy [3].

Our group previously correlated IAAs and aortic diameter in patients with thoracic aortic aneurysms between the aortic root and mid-ascending aorta [13]. We found that 69.5% of these aneurysms with abnormal IAAs, the majority associated with tri-leaflet aortic valves, would likely be denied operative intervention on account of their sub-threshold aortic diameters. In extension, the present study aimed to characterise therelationship between observed IAAs and aortic diameter in patients with BAV aortopathy to assess whether sub-threshold TAAs still harbour significant risk from an IAA >10 cm^2^/m.

## 2 Material and methods

### 2.1 Definitions

An aortic segment with diameter ≥4 cm, as measured on multi-planar contrast-enhanced computed tomography (CT) or magnetic resonance imaging (MRI), was considered aneurysmal. BAVs were identified on pre-operative *trans*-thoracic echocardiography and confirmed during surgery.

### 2.2 Patient population and study design

We performed a retrospective observational study including all consecutive adult patients undergoing first-time surgery for a BAV-related aneurysm involving the aortic root and/or ascending aorta, between 2010 and 2016 at our institution. Patient selection for surgery was primarily based on, but not limited to, the aforementioned aortic diameter cut-offs recommended in surgical guidelines, in addition to observed IAAs, cardiovascular risk factors and family and genetic history, and with consensus from an experienced aortic multi-disciplinary panel. This study has been reported in line with the STROCSS criteria [14]. The research registration unique identifying number (UIN) is NCT04756778, available at http://clinicaltrials.gov.

### 2.3 Data collection

Prospectively collected data on patient height at the time of surgery and other variables was obtained from our institutional computer database. The local ethics committee waivered the requirement for ethical approval for this retrospective study.

### 2.4 Imaging review

The multi-planar CT and/or MRI scan closest to the time of surgery was retrospectively reviewed for each patient. Maximum aortic diameters were measured using the outer edge-to-outer edge calliper technique in a plane perpendicular to blood flow at three aortic locations: the mid-point of the sinuses of Valsalva, the sino-tubular junction and the mid-ascending aorta at the level of the pulmonary artery bifurcation. The largest aortic diameter obtained from coronal and sagittal views was utilised for analysis. All imaging studies were reviewed by a single clinician with cross-checking of a randomly selected 10% sample by two experienced cardiovascular radiologists to ensure consensus in measurement technique and reproducibility.

### 2.5 Outcome measures

Median aortic diameters for all aneurysms (diameter ≥4 cm) occurring at each aortic location were calculated. Cross-sectional aortic area was calculated using the formula π x r^2^, where r represents the aortic radius (cm). This value was divided by patient height (m) to determine the cross-sectional aortic area/patient height ratio (cm^2^/m) (indexed aortic area, IAA). After identifying patients with an aneurysm at a given aortic location, we proceeded to calculate the median IAA, and proportion of at-risk patients with IAA >10 cm^2^/m, for the group of aneurysms at each aortic location. Next, aortic segments were assigned according to diameter to different sub-groups (<4.0 cm, 4.0–4.5 cm, 4.5–5.0 cm, 5.0–5.5 cm or >5.5 cm), for each of which the median IAA was calculated. Finally, the median aortic diameter corresponding to aneurysms with IAA >10 cm^2^/m was calculated at each aortic location.

### 2.6 Statistical analysis

Statistics are presented as percentages for categorical variables, and medians and ranges for continuous variables.

## 3 Results

### 3.1 Patient population

69 patients with BAVs undergoing surgery for thoracic aortic aneurysms between 2010 and 2016 at our institution were eligible for analysis. Pre-operative patient characteristics are shown in Table 1. Out of these 69 patients, aneurysms involved the mid-sinus of Valsalva in 53 (76.8%), the sino-tubular junction in 24 (34.8%) and the mid-ascending aorta in 65 (94.2%). 47/69 patients (68.1%) had a regurgitant BAV and 2/69 (2.9%) had Marfan's syndrome. Table 2 shows the distribution of aortic procedures performed.

**Table 1:** Pre-operative patient characteristics.

Characteristic	Value
Age (years)	61 (52–68)
Male	59 (85.5)
BMI	27 (24–30)
Logistic EuroSCORE	4.65 (2.23–6.37)
NYHA class	
I	22 (31.9)
II	39 (56.5)
III	8 (11.6)
Hypertension	34 (49.3)
Hypercholesterolaemia	22 (31.9)
Smoking	33 (47.8)
Pulmonary disease	4 (5.8)
CKD stage	
1	31 (44.9)
2	34 (49.3)
3	2 (2.9)
4	1 (1.4)
5	1 (1.4)
Pre-operative dialysis	1 (1.4)
TIA/CVA	4 (5.8)
Previous MI	7 (10.1)
Peripheral vascular disease	0 (0.0)
LV function	
Good	60 (87.0)
Moderate	7 (10.1)
Poor	2 (2.9)
RV function	
Good	67 (97.1)
Moderate	2 (2.9)
Poor	0 (0.0)
Marfan syndrome	2 (2.9)
Other connective tissue disease	0 (0.0)
Aortic regurgitation	47 (68.1)

Values are n (%) or median (inter-quartile range).

BMI, body mass index; CKD, chronic kidney disease; CVA, cerebrovascular accident; EuroSCORE, European System for Cardiac Operative Risk Evaluation; LV, left ventricle; MI, myocardial infarction; NYHA, New York Heart Association; RV, right ventricle; TIA, transient ischemic attack.

**Table 2:** Distribution of aortic procedures performed.

Procedure	Value
Aortic root replacement	35 (50.7)
Aortic root replacement + CABG	6 (8.70)
Ascending aortic replacement + AVR	20 (29.0)
Ascending aortic replacement + AVR + CABG	6 (8.70)
Ascending aortic replacement + arch replacement + AVR	1 (1.45)
Ascending aortic replacement + arch replacement	1 (1.45)

Values are n (%).

AVR, aortic valve replacement; CABG, coronary artery bypass graft.

### 3.2 Median aortic diameter

We identified aneurysms with diameter ≥4 cm and calculated the median diameter for these aneurysms at each aortic location, as shown in Table 3. Notably, median diameters for BAV-related TAAs were all <5.0 cm.

**Table 3:** Median aortic diameter in BAV-related thoracic aortic aneurysms according to aneurysm location.

Aneurysm location	Aortic diameter (cm)
Mid-sinus (n = 53)	4.75 (4.05–5.86)
Sino-tubular junction (n = 24)	4.43 (4.00–5.10)
Mid-ascending (n = 65)	4.90 (4.45–9.12)

Values are median (range).

### 3.3 Median indexed aortic areas and indexed aortic areas >10 cm^2^/m

Table 4 shows the median IAAs, and proportions of BAV-related TAAs with IAA >10 cm^2^/m, between the aortic root and mid-ascending aorta. Median IAAs exceeded the 10 cm^2^/m cut-off, indicating an elevated risk of aortic dissection/rupture, at both the mid-sinus and mid-ascending aorta locations at 10.1 cm^2^/m and 11.0 cm^2^/m, where 27/53 (50.9%) and 42/65 (64.6%) patients, respectively, had an abnormal IAA. Only 3/24 (12.5%) patients with aneurysms at the sino-tubular junction had an abnormal IAA, although the median IAA of 8.52 cm^2^/m here approached the 10 cm^2^/m cut-off value.

**Table 4:** Median indexed aortic areas (cm^2^/m) and proportions of aortic aneurysms with indexed aortic area >10 cm^2^/m according to aneurysm location in patients with BAV-related thoracic aortic aneurysms.

Aneurysm location	Median indexed aortic area in aneurysms cm^2^/m	Indexed aortic area >10 cm^2^/m in aneurysms N (%)
Mid-sinus	10.1 (7.25–18.3)	27/53 (50.9)
Sino-tubular junction	8.52 (5.38–11.9)	3/24 (12.5)
Mid-ascending	11.0 (6.97–40.8)	42/65 (64.6)

Values are median (range) or n (%).

Importantly, out of a total of 142 aneurysmal aortic segments in this group of 69 patients with a BAV, 72 (50.7%) were associated with an abnormal IAA.

### 3.4 Median indexed aortic areas corresponding to aortic diameter

**Fig. 1:**
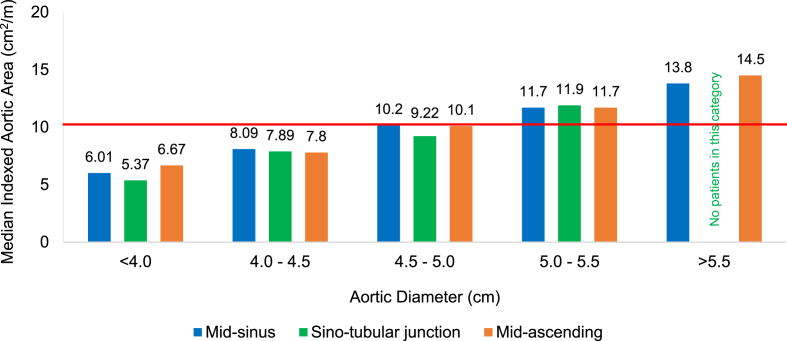
Median indexed aortic areas corresponding to aortic diameter in BAV-related thoracic aortic aneurysms (red line denotes indexed aortic area cut-off value at 10 cm^2^/m). (For interpretation of the references to colour in this figure legend, the reader is referred to the Web version of this article.)

Fig. 1 demonstrates median IAAs corresponding to aortic diameters <4.0 cm, 4.0–4.5 cm, 4.5–5.0 cm, 5.0–5.5 cm and >5.5 cm at mid-sinus, sino-tubular junction and mid-ascending aortic locations. Median IAAs increase proportionally with enlarging aortic diameter. Abnormal median IAAs surpassing the 10 cm^2^/m cut-off value, indicated by the horizontal red line, first emerge at diameters of 4.5–5.0 cm with values of 10.2 cm^2^/m and 10.1 cm^2^/m at the mid-sinus and mid-ascending aortic locations, respectively. Median IAAs within the 5.0–5.5 cm diameter range clearly surpassed the abnormal 10 cm^2^/m cut-off value, regardless of the aortic segment involved.

Table 5 shows the proportions of aneurysms with IAA >10 cm^2^/m for each aortic diameter range. There were no aneurysms with abnormal IAA corresponding to aortic diameter <4.5 cm. However, 28/54 (51.9%) aneurysms with aortic diameter 4.5–5.0 cm had an abnormal IAA, rising to 24/27 (88.9%) at 5.0–5.5 cm and 15/15 (100%) at diameter >5.5 cm. Therefore, out of a total 81 intermediate-sized aneurysms between 4.5 and 5.5 cm in diameter, a significant 52 (64.2%) had an abnormal IAA.

**Table 5:** Proportions of BAV-related thoracic aortic aneurysms with indexed aortic area >10 cm^2^/m according to aortic diameter.

Aortic diameter (cm)	N (%)	
<4.0	0/64	(0.0)
4.0–4.5	0/43	(0.0)
4.5–5.0	28/54	(51.9)
5.0–5.5	24/27	(88.9)
>5.5	15/15 (100)	

### 3.5 Relationship between indexed aortic area and aortic diameter

Aneurysms with IAA >10 cm^2^/m were grouped according to the aortic location to determine their median diameter and the proportions of individual aneurysms with diameter <5.5 cm. As shown in Table 6, median aortic diameter was <5.5 cm across all aortic locations, being 4.98 cm at the mid-sinus of Valsalva, 5.04 cm at the sino-tubular junction and 5.11 cm at the mid-ascending aorta. 22/27 (81.5%), 3/3 (100%) and 32/42 (68.6%) of aneurysms with an abnormal IAA involving the mid-sinus of Valsalva, sino-tubular mid-ascending aorta, respectively, concurrently had a diameter <5.5 cm.

**Table 6:** Relationship between indexed aortic area ranges and median aortic diameters in BAV-related thoracic aortic aneurysms.

	Aneurysm location		
	Mid-sinus N = 27	Sino-tubular junction N = 3	Mid-ascending N = 42
Median aortic diameter cm	4.98 (4.62–6.47)	5.04 (5.03–5.10)	5.11 (4.63–9.12)
Aneurysms with diameter <5.5 cm N (%)	22/27 (81.5)	3/3 (100)	32/42 (68.8)

Values are median (range) or n (%).

Considering the entire group of 72 BAV-related aneurysms with an IAA >10 cm^2^/m, a significant 57 (79.2%) had an aortic diameter <5.5 cm. In the absence of any valvular indication for surgery, they would not fulfil the size criteria indicating aortic surgery recommended in current guidelines, albeit being subject to an elevated risk of aortic complications. Under strict interpretation of these recommendations, surgical intervention would only be applicable in 15/72 aortic segments (20.8%) in this BAV population with both abnormal IAA and aortic diameter >5.5 cm.

## 4 Discussion

This is the first study to evaluate the relationship between aortic diameter and cross-sectional aortic area/patient height ratio (indexed aortic area, IAA) exclusively in patients with BAV aortopathy. We employed an abnormal IAA >10 cm^2^/m as a size-based determinant of increased risk to identify those aneurysms susceptible to aortic dissection/rupture, especially those with smaller aortic diameters than are recommended for surgical resection. Importantly, we did not seek to quantify the exact risks associated with an IAA >10 cm^2^/m, compare outcomes between surgical intervention and medical management of BAV aortopathy at any particular diameter interval, and neither did we aim to assess the incidence of aortic adverse events, namely dissection, rupture and re-intervention, in our study population.

Our results confirm that (i) IAA can be utilised effectively to identify those aneurysms at increased risk of dissection/rupture attributed to IAA >10 cm^2^/m, (ii) large proportions of patients at sub-threshold aortic diameters of 4.5–5.0 cm possess abnormal IAAs and (iii) large proportions of BAV-related proximal aortic aneurysms remain are at increased risk of these adverse aortic events but may not be selected for surgery owing to diameter <5.5 cm.

Studies analysing indexed measurements of aortic size in BAV patients as pertains to the risk of aortic adverse events and selection for preventative aortic surgery are limited in literature. Only two relevant papers were retrieved from a search on the PubMed database using the terms “cross-sectional aortic area AND bicuspid”. Svensson and colleagues operated on 40 patients with BAV-associated acute and chronic aortic dissections and reported that 35% had aortic diameters <5.5 cm, with a mean IAA of 18.34 ± 8.88 cm^2^/m [8]. Masri and colleagues evaluated IAA in asymptomatic patients with BAV-related TAAs, observing that the IAA was abnormal in 33% of 969 patients with a dilated proximal aorta in conjunction with a BAV [11]. Furthermore, they identified 64% of 405 patients, and 43% of 256 patients with a 4.5–5.5 cm “intermediate-sized” ascending aorta or aortic root, respectively, had an IAA exceeding the 10 cm^2^/m cut-off [11].

BAV represents the most common congenital heart defect prevalent in approximately 1–2% of adults [15,16]. The potential adverse sequelae, including aortic valve dysfunction, endocarditis, aortic coarctation, aneurysm formation and acute dissection or rupture, render bicuspid aortic valvulopathy accountable for more deaths than all other congenital heart lesions combined [17]. Proximal aortic dilatation affects around 40–60% of patients with BAV aortopathy irrespective of valve dysfunction [18,19], and both aortic expansion and valvular stenosis progress faster in association with a BAV [[20], [21], [22]]. Aortic dissection occurs at an earlier age in patients with BAV, in whom the risks of aortic dissection and rupture remain 6- to 9-fold greater than in the general population [23,24].

Recently there has been greater emphasis on elucidating the natural history of BAV disease to garner support for its more aggressive treatment on account of its potentially life-threatening complications. Yet despite advancements in the understanding of the pathophysiological mechanisms and genetic basis of BAV aortopathy in recent years, in addition to the implications of aortic dimensions, valve phenotype and ascending aortic morphology [13,[25], [26], [27], [28], [29], [30]], there is conflicting evidence on the optimal timepoint for surgical intervention on proximal TAAs in patients with BAV. In the absence of a valvular indication and coexisting risk factors, guidelines justify surgery in this group at aortic diameters ≥5.5 cm [[1], [2], [3]]. However, patients undergoing isolated BAV replacement with accompanying ascending aortic diameter <5.0 cm are nevertheless significantly vulnerable to future complications in the non-intervened aorta [31,32].

In the present study, we demonstrate that 57/72 (79.2%) BAV-associated aneurysms with an abnormal IAA might not be eligible for aortic repair despite their greater susceptibility to aortic dissection, since they fall short of the diameter thresholds at which aneurysm surgery is proposed in current management guidelines. Abnormal IAAs first become apparent in BAV-related TAAs at aortic diameters of 4.5–5.0 cm, where over half of aneurysms had an IAA >10 cm^2^/m. By the time the aorta has acquired a 5.0–5.5 cm diameter, IAAs are abnormal in over 88% of patients with a BAV. This is in concurrence with previous work from our group showing that 49.1% and 98.5% of dilated thoracic aortas measuring 4.5–5.0 cm and 5.0–5.5 cm, respectively, possessed abnormal IAAs in a mixed, predominantly tri-leaflet, aortic valve population [13].

Median aortic diameters for aneurysms at the mid-sinus, sino-tubular junction and mid-ascending aortic locations were all smaller than the 5.5 cm threshold advocated for aortic replacement, and in fact, fewer than 5 cm. Despite this, median IAAs were 10.1 cm^2^/m at the mid-sinus, where IAA in over 50% of aneurysms surpassed the 10 cm^2^/m limit indicative of increased aortic complication risks, and 11.0 cm^2^/m at the mid-ascending aorta, where almost 65% of patients had an increased IAA. At the sino-tubular junction, median IAAs in BAV-related aneurysms approached an abnormal value at 8.52 cm^2^/m, but only 12.5% of individual aneurysms at this location had an elevated IAA.

Whilst contemporary guidelines endorse replacement of the aneurysmal thoracic aorta in the presence of a functional BAV once a critical diameter of 5.5 cm is reached [[1], [2], [3]], it is evident from the present study that IAAs at this aortic diameter are highly abnormal, and it may therefore be inappropriate to delay surgery until the aorta has attained this size. Many patients could experience a lethal aortic event once their IAA exceeds 10 cm^2^/m, but before even reaching a 5.5 cm aortic diameter. Indeed, at diameters >5.5 cm, all patients with BAV in our series had a markedly abnormal IAA, ranging from 14.3 cm^2^/m at the mid-sinus to 40.8 cm^2^/m at the mid-ascending aorta.

Our important findings suggest that intervening on patients with bicuspid valve aortopathy at the present 5.5 cm diameter cut-off, in the absence of additional risk factors, may not be sufficient to prevent the onset of potentially disastrous aortic events. The occurrence of abnormal IAA even at diameter ranges of 4.5–5.0 cm nevertheless implies significant aortic risk. Further work is necessary to ascertain whether IAA may be a better size-based determinant for surgery than absolute diameter in BAV populations, and the optimal size interval at which aortic replacement should be performed in this high-risk category. Fundamental to patient selection for aortic surgery is careful consideration of the potential hazards and intended benefits of the planned procedure on an individual patient basis. Whilst dedicated aortic centres demonstrate excellent and reproducible long-term results, the operative risks of stroke, endocarditis and graft infection must be judiciously balanced against the accruing annual risks of dissection/rupture in those undergoing surveillance without definitive surgical treatment.

The main strength of this study is that it represents the first analysis to correlate indexed aortic area, as a validated and alternative indicator of aortic dimensions, with absolute aortic diameter in proximal thoracic aortic aneurysms associated exclusively with a BAV. In this special population, we identifiy susceptible aneurysms which might otherwise not be deemed to be at such a significant risk of aortic complications to warrant intervention, on the basis of their sub-threshold aortic diameter.

The retrospective, observational nature of this study implies potential for referral and selection bias. Whilst our BAV study population was relatively small, it is plausible that the key findings obtained would not be significantly altered by simply increasing the pool of patients analysed. Utilising the formula π x r^2^ for aortic area calculations may incorrectly assume a circular cross-section to the aorta, whereas a planimetry technique may be more precise. Furthermore, by design, it is beyond the scope of this study to recommend a more conservative diameter threshold for the surgical resection of BAV-related aneurysms than that documented in current guidelines, since this would mandate prospective analyses incorporating outcome data from extended longitudinal follow-up.

## 5 Conclusion

Significant proportions of patients with a BAV and proximal aortic aneurysms have abnormal IAAs >10 cm^2^/m, indicating their increased risk of dissection or rupture, associated with sub-threshold aortic diameters that do not satisfy the size criteria justifying surgical intervention proposed in international guidelines. The role of the IAA parameter requires further assessment to clarify whether patients with BAV-related aortopathy warrant more proactive surgery at smaller aortic diameters than are currently recommended.

## Ethical approval

Requirement for ethical approval waivered by local ethics committee.

## Sources of funding

Nothing to declare.

## Author contribution

Metesh Acharya: Conceptualization, Writing – original draft, Writing – review & editing, Methodology, Investigation, Formal analysis, Visualization. Oswaldo Valencia: Investigation, Formal analysis. Mark Edsell: Writing – review & editing. Maite Tome: Writing – review & editing. Robert Morgan: Writing – review & editing. Justin Nowell: Writing – review & editing. Marjan Jahangiri: Conceptualization, Writing – review & editing, Visualization, Supervision, Project Administration.

## Conflicts of interest

Nothing to declare.

## Research registration unique identifying number (UIN)

Name of the registry: ClinicalTrials.gov.

Unique Identifying number or registration ID: NCT04756778.

Hyperlink to your specific registration (must be publicly accessible and will be checked): Study Link.

## Guarantor

Metesh Acharya.

## Provenance and peer review

Not commissioned, externally peer reviewed.

## References

[1] Erbel R., Aboyans V., Boileau C., Bossone E., Bartolomeo R.D., Eggebrecht H. (2014). ESC Guidelines on the diagnosis and treatment of aortic diseases: document covering acute and chronic aortic diseases of the thoracic and abdominal aorta of the adult. The Task Force for the Diagnosis and Treatment of Aortic Diseases of the European Society of Cardiology (ESC). Eur. Heart J..

[2] Hiratzka L.F., Bakris G.L., Beckman J.A., Bersin R.M., Carr V.F., Casey D.E. (2010). ACCF/AHA/AATS/ACR/ASA/SCA/SCAI/SIR/STS/SVM guidelines for the diagnosis and management of patients with thoracic aortic disease: a report of the American College of Cardiology foundation/American heart association task force on practical guidelines, American association for thoracic surgery, American College of radiology, American stroke association, society of cardiovascular anesthesiologists, society for cardiovascular angiography and interventions, society of interventional radiology, society of thoracic surgeons, and society for vascular medicine. Circulation.

[3] Hiratzka L.F., Nishimura R.A., Bonow R.O., Creager M.A., Guyton R.A., Isselbacher E.M. (2016). Surgery for aortic dilatation in patients with bicuspid aortic valves: a statement of clarification from the American College of Cardiology/American heart association task force on clinical practice guidelines. Circulation.

[4] Otto C.M., Nishimura R.A., Bonow R.O., Carebello B.A., Erwin J.P., Gentile F. (2020). ACC/AHA guideline for the management of patients with valvular heart disease: executive summary: a report of the American College of Cardiology/American heart association joint committee on clinical practice guidelines. Circulation.

[5] Pape L.A., Tsai T.T., Isselbacher E.M., Oh J.K., O'Gara P.T., Evangelista A. (2007). Aortic diameter >or = 5.5 cm is not a good predictor of type A aortic dissection: observations from the International Registry of Acute Aortic Dissection (IRAD). Circulation.

[6] Svensson L.G., Khitin L. (2002). Aortic cross-sectional area/height ratio and timing of aortic surgery in asymptomatic patients with Marfan syndrome. J. Thorac. Cardiovasc. Surg..

[7] Isselbacher E.M., Lino Cardenas C.L., Lindsay M.E. (2016). Hereditary influence in thoracic aortic aneurysms and dissection. Circulation.

[8] Svensson L.G., Kim K.H., Lytle B.W., Cosgrove D.M. (2003). Relationship of aortic cross-sectional area to height ratio and the risk of aortic dissection in patients with bicuspid aortic valves. J. Thorac. Cardiovasc. Surg..

[9] Davies R.R., Kaple R.K., Mandapati D., Gallo A., Botta D.M., Elefteriades J.A. (2007). Natural history of ascending aortic aneurysms in the setting of an unreplaced bicuspid aortic valve. Ann. Thorac. Surg..

[10] Masri A., Kalahasti V., Svensson L.G., Roselli E.E., Johnston D., Hammer D. (2016). Aortic cross-sectional area/height ratio and outcomes in patients with a trileaflet aortic valve and a dilated aorta. Circulation.

[11] Masri A., Kalahasti V., Svensson L.G., Alashi A., Schoenhagen P., Roselli E.E. (2017). Aortic cross-sectional area/height ratio and outcomes in patients with bicuspid aortic valve and a dilated ascending aorta. Circ. Cardiovasc. Imaging.

[12] Wojnarksi C.M., Svensson L.G., Roselli E.E., Idrees J.J., Lowry A.M., Ehrlinger J. (2015). Aortic dissection in patients with bicuspid aortic valve-associated aneurysms. Ann. Thorac. Surg..

[13] Acharya M.N., Youssefi P., Soppa G., Valencia O., Nowell J., Kanagasabay R. (2018). Analysis of aortic area/height ratio in patients with thoracic aortic aneurysm and Type A dissection. Eur. J. Cardio. Thorac. Surg..

[14] Agha R., Abdall-Razak A., Crossley E., Dowlut N., Iosifidis C., Mathew G., Strocss Group, Strocss (2019). Guideline: strengthening the reporting of cohort studies in surgery. Int. J. Surg..

[15] Braverman A.C., Güven H., Beardslee M.A., Makan M., Kates A.M., Moon M.R. (2005). The bicuspid aortic valve. Curr. Probl. Cardiol..

[16] Hoffman J.I., Kaplan S. (2002). The incidence of congenital heart disease. J. Am. Coll. Cardiol..

[17] Ward C. (2000). Clinical significance of the bicuspid aortic valve. Heart.

[18] Ram D., Bouhout I., Karliova I., Schneider U., El-Hamamsy I., Schäfers H.J. (2020). Concepts of bicuspid aortic valve repair: a review. Ann. Thorac. Surg..

[19] Wald O., Korach A., Shapira O.M. (2010). Should aortas in patients with bicuspid aortic valve really be resected at an earlier stage than tricuspid?. PRO. Cardiol. Clin..

[20] Beppu S., Suzuki S., Matsuda H., Ohmori F., Nagata S., Miyatake K. (1993). Rapidity of progression of aortic stenosis in patients with congenital bicuspid aortic valves. Am. J. Cardiol..

[21] Cannata A., Russo C.F., Vitali E. (2008). Bicuspid aortic valve: about natural history of ascending aorta aneurysms. Ann. Thorac. Surg..

[22] Tadros T.M., Klein M.D., Shapira O.M. (2009). Ascending aortic dilatation associated with bicuspid aortic valve: pathophysiology, molecular biology, and clinical implications. Circulation.

[23] Tzemos N., Therrien J., Yip J., Thanassoulis G., Tremblay S., Jamorski M.T. (2008). Outcomes in adults with bicuspid aortic valves. J. Am. Med. Assoc..

[24] Michelena H.I., Khanna A.D., Mahoney D., Margaryan E., Topilsky Y., Suri R.M. (2011). Incidence of aortic complications in patients with bicuspid aortic valves. J. Am. Med. Assoc..

[25] Della Corte A., Bancone C., Buonocore M., Dialetto G., Covino F.E., Manduca S. (2013). Pattern of ascending aortic dimensions predicts the growth rate of the aorta in patients with bicuspid aortic valve. JACC Cardiovasc. Imaging.

[26] Della Corte A., Michelena H.I., Citarella A., Votta E., Piatti F., Lo Presti F. (2019). Risk stratification in bicuspid aortic valve aortopathy: emerging evidence and future perspectives. Curr. Probl. Cardiol..

[27] Jackson V., Olsson C., Eriksson P., Franco-Cereceda A. (2013). Aortic dimensions in patients with bicuspid and tricuspid aortic valves. J. Thorac. Cardiovasc. Surg..

[28] Pederson M.W., Groth K.A., Mortensen K.H., Brodersen J., Gravholt C.H., Andersen N.H. (2019). Clinical and pathophysiological aspects of bicuspid aortic valve disease. Cardiol. Young.

[29] Andreassi M.G., Della Corte A. (2016). Genetics of bicuspid valve aortopathy. Curr. Opin. Cardiol..

[30] Masri A., Svensson L.G., Griffin B.P., Desai M.Y. (2017). Contemporary natural history of bicuspid aortic valve disease: a systematic review. Heart.

[31] Borger M.A., Preston M., Ivanov J., Fedak P.W., Davierwala P., Armstrong S. (2004). Should the ascending aorta be replaced more frequently in patients with bicuspid aortic valve disease?. J. Thorac. Cardiovasc. Surg..

[32] Verma S., Siu S.C. (2014). Aortic dilatation in patients with bicuspid aortic valve. N. Engl. J. Med..

